# Supplementary material for: Short-Term Exposure to Benzo(a)Pyrene Causes Disruption of GnRH Network in Zebrafish Embryos
Source: Int J Mol Sci. 2023 Apr 7;24(8):6913. doi: 10.3390/ijms24086913 (PMC10138490; doi:10.3390/ijms24086913)
Supplement: Supplementary file 1 [file ijms-24-06913-s001.zip › ijms-2273968-supplementary.pdf]

Supplementary Material

Short-Term Exposure to Benzo(a)Pyrene Causes Disruption of GnRH Network in Zebrafish Embryos

Ilaria Gentile [1], Valeria Vezzoli [2], Sara Martone [3], Maria Grazia Totaro [3], Marco Bonomi [1,2], Luca Persani [1,2,*] and Federica Marelli [2]

[1] Department of Medical Biotechnology and Translational Medicine, University of Milan, 20133 Milan, Italy; ilaria.gentile@unimi.it (I.G.); m.bonomi@auxologico.it (M.B.)
[2] Division of Endocrine and Metabolic Diseases, Laboratory of Endocrine and Metabolic Research, IRCCS Istituto Auxologico Italiano, 20149 Milan, Italy; v.vezzoli@auxologico.it (V.V.); federica.marelli.fm@gmail.com (F.M.)
[3] IFOM-FIRC, Institute of Molecular Oncology, 20139 Milan, Italy; sara.martone@ifom.eu (S.M.); mariagrazia.totaro@ifom.eu (M.G.T.)
* Correspondence: luca.persani@unimi.it; Tel.: +39-02619112432

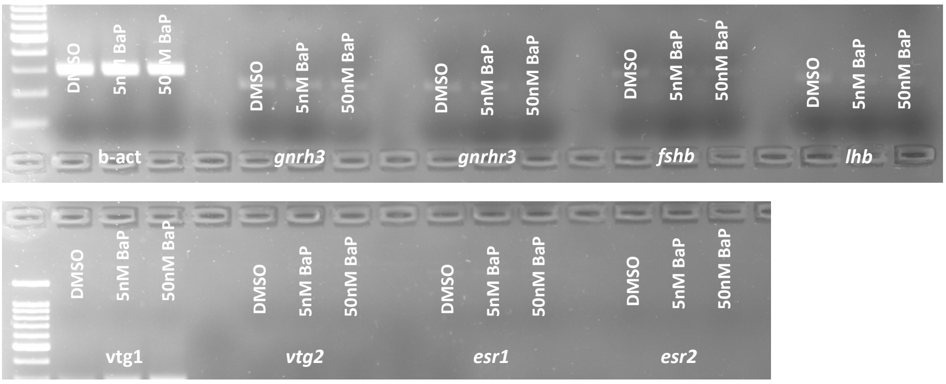

Supplementary Figure S1: Electrophoresis of qPCR products of *b-actin*, *gnrh3*, *gnrhr3*, *fshb*, *lhb*, *vtg1*, *vtg2*, *esr1*, and *esr2*. Representative qPCR samples were run on 3% agarose gel and acquired using c600 Azure Imaging System (Azure Biosystems).

Supplementary Table S1: List of qPCR primers used in this study

Gene (NCBI geneID/ NM)	Primer Sequence (5′-3′)
b-actin<br>(ID: 57934 / NM_131031.1)	*b-act*-Fw: GTGCTAGACTCTGGTGATGGTGTG<br>*b-act*-Rv: GGTGATGACCTGACCTGACCGTC
sod1<br>(ID: 30553 / NM_131294.1)	*sod1*-Fw: GGCCAACCGATAGTGTGAGA<br>*sod1*-Rv: GAGTCAGCATTGCATCCTCG
sod2<br>(ID: 335799 / NM_199976.1)	*sod2*-Fw: TCATCACAGCAAGCACCATG<br>*sod2*-Rv: GGTTGTCACATCACCCTTGG
cat<br>(ID: 30068 / NM_130912.2)	*cat1*-Fw: CGATGACAACGTGACCCAAG<br>*cat1*-Rv: TTCAGATGCCCGGCCATATT
ogg1<br>(ID: 158858 / NM_001123308.2)	*ogg1*-Fw: CTTCACAGGAGTGCGTTTGC<br>*ogg1*-Rv: AGTCTTTCGACCATGCCCTG
casp3a<br>(ID: 140621 / NM_131877.3)	*casp3a*-Fw: AGGCTTGTCGAGGAACAGAA<br>*casp3a*-Rv: CTGGGATCCTTACACGACCA
casp3b<br>(ID:566604 / NM_131889.4)	*casp3b*-Fw: TCTGGCATTGAGTGTGACGG<br>*casp3b*-Rv: TATCCTGGTGCGGTGGAGTA

Gene (NCBI geneID/ NM)	Primer Sequence (5′-3′)
fshβ<br>(ID: 402919 / NM_205624.1)	*fshβ*-Fw: GGACTATGCTGGACAATGGATC<br>*fshβ*-Rv: ACAGCTCAGAGCCACGGG
lhβ<br>(ID: 402917 / NM_205622.2)	*lhβ*-Fw: TGTTATTGGCTGGAAATGGTGTC<br>*lhβ*-Rv: CGGGCTCTTGTAAACGGGAT
esr1<br>(ID: 259252 / NM_152959.1)	*esr1*-Fw: CTGGAAGTGTTGATGATTGGC<br>*esr1*-Rv: ATGCACCAGAATTGATGAGTATG
esr2a<br>(ID: 317734 / NM_180966.2)	*esr2*-Fw: CGACTCCGCAAGTGCTATGA<br>*esr2*-Rv: CGCCTCTAGAATACAGCTAACCA
vtg1<br>(ID: 559475 / NM_001044897.3)	*vtg1*-Fw: TCACTGCTGATCCTGAAACCAT<br>*vtg1*-Rv: GGTGACCAGCATTGCCCA
vtg2<br>(ID: 559931 / NM_001044913.1)	*vtg2*-Fw: GACATTCTCAAGGGCAACTACAAG<br>*vtg2*-Rv: GAGCAGGAGCTTTCACAGGAGT
gnrhr3<br>(ID: 564687 /NM_001177450.1)	*gnrhr3*-Fw: CGGCAGTTTCAGCGAGC<br>*gnrhr3*-Rv: GGACAGCACAATGATGAGGG
